# Association of *CACNG6 *polymorphisms with aspirin-intolerance asthmatics in a Korean population

**DOI:** 10.1186/1471-2350-11-138

**Published:** 2010-09-23

**Authors:** Jin Sol Lee, Jeong-Hyun Kim, Joon Seol Bae, Jason Yongha Kim, Tae Joon Park, Charisse Flerida Pasaje, Byung-Lae Park, Hyun Sub Cheong, Soo-Taek Uh, Jong-Sook Park, An-Soo Jang, Mi-Kyeong Kim, Inseon S Choi, Choon-Sik Park, Hyoung Doo Shin

**Affiliations:** 1 Department of Life Science, Sogang University, Seoul 121-742, Republic of Korea; 2 Department of Genetic Epidemiology, SNP Genetics, Inc., Seoul 153-801, Republic of Korea; 3 Division of Allergy and Respiratory Medicine, Soonchunhyang University Seoul Hospital, Seoul 140-743, Republic of Korea; 4 Genome Research Center for Allergy and Respiratory Disease, Soonchunhyang University Bucheon Hospital, Bucheon 420-767, Republic of Korea; 5 Division of Internal Medicine, Chungbuk National University, College of Medicine, Cheongju 361-711, Republic of Korea; 6 Department of Allergy, Chonnam National University Medical School and Research Institute of Medical Sciences, Gwangju 501-757, Republic of Korea

## Abstract

**Background:**

Aspirin-intolerant asthma (AIA) occurs in the lower and upper airways through excessive production of leukotrienes upon administration of non-steroidal anti-inflammatory drugs (NSAIDs). One of the three symptoms of AIA is nasal polyposis, a chronic inflammatory disease that is related to the function of calcium ion in recruitment of immune cells during airway inflammation. It has been implicated that bronchodilation in the airway is related to Ca(2+) regulation. The *calcium channel, voltage-dependent, gamma subunit 6 *(*CACNG6*) gene encodes a protein that stabilizes the calcium channel.

**Methods:**

To study the associations between AIA and polymorphisms in *CACNG6 *gene, eight variants were genotyped in 102 AIA cases and 429 aspirin-tolerant asthma (ATA) controls. Logistic analyses were used to evaluate the associations of *CACNG6 *polymorphisms with AIA.

**Results:**

Statistical analyses revealed that a single nucleotide polymorphism (SNP; *rs192808C > T*; *P *= 0.0004, *P^corr ^*= 0.0029, OR = 2.88 in co-dominant model; *P *= 0.0005, *P^corr ^*= 0.0036, OR = 2.99 in dominant model) in intron and a haplotype unique to this variant (*CACNG6_BL1_ht6*; *P *= 0.003, *P^corr ^*= 0.02, OR = 2.57 in co-dominant model, *P *= 0.001, *P^corr ^*= 0.0087, OR = 2.81 in dominant model) were significantly associated with the risk of AIA.

**Conclusions:**

Our results suggest that the *CACNG6 *variants might be associated with the risk of AIA in a Korean population.

## Background

Aspirin-intolerant asthma (AIA), which was first described in 1922, is a unique clinical syndrome associated with acute bronchconstriction upon administration of aspirin and other non-steroidal anti-inflammatory drugs [1]. Although considered as one of the most widely used medication, aspirin intake also incurs various side effects including manifested gastrointestinal ulcer, stomach bleeding and tinnitus especially in higher doses. Although the side effects caused by aspirin are not common, these effects have been reported in about 10% of adult asthmatics.

The triad of AIA symptoms includes aspirin sensitivity, bronchial asthma and chronic rhinosinusitis with nasal polyposis [2-4]. Nasal polyposis is a chronic inflammatory disease of the upper respiratory tract that affects around two thirds of patients with AIA. The polyps are filled with mast cells and eosinophils due to high levels of the proinflammatory cysteinyl leukotrienes (cysLTs) [5]. After mast cells are activated, it releases a variety of paracrine signals that target tissues such as bronchi and vasculature to recruit other immune cells at the inflammatory site. Among the remarkable signaling molecules released by mast cells, the cysLTs that includes leukotriene C(4) (LTC4), LTD4, and LTE4 are well known [6,7]. The LTC4 is secreted from mast cells following Ca(2+) influx through store-operated calcium release-activated calcium (CRAC) channels [7-10]. It has also been reported that proteinase activated receptor-2 (PAR2), as a novel emerging pharmacological target in airway diseases, is expressed in the airway epithelium and its activation may be involved in the protective role of preserving bronchial functionality [11,12]. In line with the fact that the airway smooth muscle cell contraction is regulated by changes in intracellular Ca(2+) concentration, a previous molecular report has revealed a relationship between this Ca(2+) regulation and bronchodilation in the airway [13]. More recently, novel actions of NSAIDs on vascular ion channels including L-type calcium channel has also been suggested [14].

The L-type calcium channels are composed of five subunits. The *CACNG6 *gene encodes one of these subunits, specifically a gamma subunit protein which was first identified in muscle cells [15]. Recent studies have revealed negative relations of *CACNG6 *gene expression to chronic obstructive pulmonary disease, responses of the human airway epithelium following injury and % parenchyma in lung tissues [16,17]. The CACNG6 is an integral membrane protein that stabilizes the calcium channel during its inactive state [18]. Since the role of azelastine in asthma treatment is achieved by blocking the L-type calcium channel thus, preventing Ca(2+) current [19], the CACNG6 may, therefore, be of interest in the study of asthma treatment. Moreover, abnormal Ca(2+) influx may induce leukotriene overproduction from mast cells, leading to recruitment of other immune cells, such as eosinophils, resulting in vasospasm by eosinophilic infiltration of the coronary artery wall and cardiac contractility [8,20]. Although many signaling pathways have been known to underlie the reversible contraction of airway smooth muscle, ionic mechanisms related to Ca(2+) and the calcium channels in the airways are still poorly understood [21]. In this connection, we explored whether *CACNG6 *single nucleotide polymorphisms (SNPs) are associated with AIA.

## Methods

### Study Subjects

Subjects in this study were recruited from the Asthma Genome Research Center comprising hospitals of Soonchunhyang, Chunnam, Chungbuk, Seoul National and Chung-Ang Universities in Korea. All subjects provided written informed consents and the study protocols were approved by the Institutional Review Board of each hospital. Diagnosis of AIA was performed according to a modified method as previously described [22]. We also performed aspirin challenge in subjects with a history of aspirin hypersensitivity, presence of urticaria, nasal polyps and sinusitis. The AIA case group included patients with 20% or greater decreases in FEV_1 _or 15% to 19% decreases in FEV_1 _with naso-ocular or cutaneous reactions, whereas subjects showing a rate of FEV_1 _decline less than 15% without extrabronchial, nasal or skin symptoms were included in ATA group.

### SNP selection and genotyping

We selected 8 common polymorphic SNPs from the International HapMap Project phase 1&2 http://hapmap.ncbi.nlm.nih.gov/ and National Center for Biotechnology Information (build 36) based on the minor allele frequency (> 0.05) and LD status. Among three SNPs (*rs3810244*, *rs158194*, *rs4806481*) in LD (|D'| > 0.7), the most common SNP (*rs4806481*) was selected for genotyping. For examination of AIA risk association, we genotyped 8 common SNPs in the *CACNG6 *gene. Genotyping was carried out with 20 ng of genomic DNA by TaqMan assay in the ABI prism 7900HT sequence detection system software version 2.3 (Applied Biosystems, CA, USA) in 102 AIA cases and 429 ATA controls with the assessment of data quality by duplicate DNAs (n = 10). A total of 8 polymorphisms on *CACNG6 *were successfully genotyped.

### Statistics

We calculated linkage disequilibrium to all pairs of biallelic loci using Lewontin's D' (|D'|) [23] and *r^2^*. PHASE algorithm (ver. 2.0) developed by Stephens *et al. *was used for inferring haplotypes [24]. Associations of genotypes and haplotypes in the *CACNG6 *gene with AIA were calculated using logistic analyses adjusted for age, gender, smoking status, atopy and body mass index as covariates. We also performed linear regression analysis to determine the differences in the rates of decline in FEV_1 _following aspirin challenge among the genotypes and haplotypes. The data were adjusted, managed and analyzed using SAS version 9.1 (SAS Inc., Cary, NC). The effective number of independent marker loci (7.28) was calculated for multiple testing corrections using SNPSpD software http://genepi.qimr.edu.au/general/daleN/SNPSpD/, which is based on the spectral decomposition (SpD) of matrices of pair-wise LD between SNPs.

## Results

In this study, the clinical characteristics of the study subjects showed that the fall rate by aspirin provocation in AIA patients was significantly higher than that of ATA controls (33.59 ± 13.42% vs. 3.54 ± 4.85%, respectively; *P *< 0.0001). This was in contrast to the current smoker ratio and mean age which was higher in ATA controls than those of AIA patients (21.36% vs. 30.07% for smoking status and 42.76 year vs. 47.30 year for mean age, respectively). Body mass index (BMI) and PC20 methacholine test also showed significant signals (*P *< 0.01). In addition, AIA patients were more sensitive to methacholine than ATA patients (4.26 mg/ml vs. 6.91 mg/ml). Analyses on other factors in subject's clinical characteristics showed no significant differences between AIA and ATA groups. The clinical characteristics of the study subjects are summarized in Table 1.

**Table 1:** Clinical profiles of aspirin intolerance asthma and control subjects

Clinical profile	AIA	ATA
Number of subjects (n)	102	429
Age [year, mean (range)]	42.76 (18.66-72.73)*	47.30 (15.40-77.88)
Sex (n, male/female)	37/65	147/282
Smoker (current smoker/exsmoker) (%)	21.36 (13.59/7.77)*	30.07 (12.35/17.72)
Height [cm, mean (range)]	161.70 ± 9.91	160.42 ± 8.39
Weight (kg)	61.64 ± 10.39	63.40 ± 10.97
Body mass index (kg/m^2^)	23.56 ± 3.37*	24.58 ± 3.39
% decline of FEV_1 _by aspirin provocation	33.59 ± 13.42**	3.54 ± 4.85
Blood eosinophil (%)	6.65 ± 5.78	6.03 ± 5.92
FEV_1 _(% predicted)	85.10 ± 16.41*	91.66 ± 16.87
PC20 methacholine (mg/ml)	4.26 ± 7.62*	6.91 ± 8.90
Total IgE (IU/ml)	415.74 ± 714.70	361.00 ± 607.56
Positive rate of skin test (%)	48.04	57.81

Age indicates a first medical examination. AIA, aspirin-intolerant asthma; ATA, aspirin-tolerant asthma.

*P < 0.05; **P < 0.0001 compared to ATA control.

**Figure 1:**
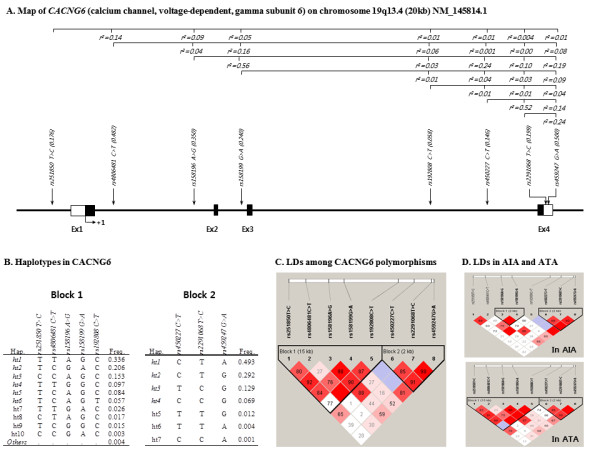
**Gene maps and haplotypes of the *CACNG6 *gene**. Schematic physical map, haplotypes and LD plot of *CACNG6*. (A) Polymorphisms identified in *CACNG6*. Coding exons are marked by shaded blocks and 3'-untranslated region (UTR) by white blocks. The LD coefficients (*r*^2^) are based on the genotypes of Korean samples. (B) Haplotypes of *CACNG6 *in the Korean population. Only those with frequencies over 0.05 are shown in Table 2 and 3. (C) LD coefficients (|D'|) among the selected SNPs based on the genotypes of whole study subjects in this study. (D) LD coefficients (|D'|) in AIA and ATA.

To investigate the association of *CACNG6 *polymorphisms with AIA, 8 SNPs (*rs251850*, *rs4806481*, *rs158196*, *rs158199*, *rs192808*, *rs450227*, *rs2291068*, *rs459247*) in the *CACNG6 *gene were genotyped in a total of 531 study subjects including 102 AIA patients as a case group and 429 ATA patients as controls. The genotype distributions of all loci were in Hardy-Weinberg equilibrium (Table 2, *P *> 0.05). Genetic map and location of *CACNG6 *polymorphisms are shown in Fig. 1A. Logistic analysis was used to analyze whether the genetic polymorphisms were associated with AIA. Interestingly, analysis revealed that a SNP in intron (*rs192808C > T*; OR = 2.88, 95% CI = 1.60-5.17, *P *= 0.0004, *P^corr ^*= 0.0029 in co-dominant model), which was more frequent in AIA patients than in ATA controls (0.103 in AIA patients and 0.045 in ATA patients) (Table 2), was significantly associated with the risk of AIA even after multiple testing corrections. In additional analysis using effective models, this allelic variant also showed a susceptibility to AIA in dominant model (OR = 2.99, 95% CI = 1.62-5.54, *P *= 0.0005, *P^corr ^*= 0.0036).

**Table 2:** SNPs information and logistic analysis for *CACNG6*

SNP/Haplotype	Allele change	Position	Frequency	Hetero-zygosity	Frequency^a^		HWE		Co-dominant			Dominant			Recessive		
								
					AIA	ATA	AIA	ATA	OR(95%CI)	*P*	*P^corr^*	OR(95%CI)	*P*	*P^corr^*	OR(95%CI)	*P*	*P^corr^*
*rs251850*	T > C	Promoter	0.176	0.29	0.176	0.177	0.904	0.139	0.97 (0.64-1.49)	0.9	-	0.93(0.58-1.49)	0.75	-	1.49(0.38-5.94)	0.57	-
*rs4806481*	C > T	Intron	0.482	0.499	0.451	0.488	0.766	0.461	0.89(0.64-1.22)	0.45	-	0.84(0.52-1.37)	0.49	-	0.86(0.50-1.49)	0.6	-
*rs158196*	A > G	Intron	0.35	0.455	0.363	0.348	0.499	0.537	0.99(0.72-1.37)	0.96	-	0.95(0.60-1.49)	0.82	-	1.08(0.57-2.04)	0.81	-
*rs158199*	G > A	Intron	0.24	0.364	0.255	0.233	0.474	0.124	1.03(0.73-1.46)	0.87	-	1.03(0.66-1.62)	0.88	-	1.05(0.45-2.41)	0.92	-
*rs192808*	C > T	Intron	0.058	0.11	0.103	0.045	0.931	0.899	2.88(1.60-5.17)	**0.0004**	**0.0029**	2.99(1.62-5.54)	**0.0005**	**0.0036**	6.11(0.28-131.25)	0.25	-
*rs450227*	C > T	Intron	0.146	0.25	0.167	0.145	0.552	0.444	1.1(0.71-1.70)	0.67	-	1.13(0.69-1.83)	0.63	-	0.98(0.19-5.01)	0.98	-
*rs2291068*	T > C	Exon	0.199	0.319	0.225	0.192	0.645	0.562	1.08(0.73-1.58)	0.71	-	1.04(0.66-1.64)	0.88	-	1.43(0.52-3.96)	0.49	-
*rs459247*	G > A	Exon	0.5	0.5	0.426	0.515	0.558	0.542	0.75(0.55-1.03)	0.07	0.54	0.66(0.41-1.07)	0.09	0.68	0.71(0.41-1.22)	0.21	-
*CACNG6_BL1_ht1*			0.339	0.448	0.324	0.338	0.884	0.665	0.97(0.70-1.36)	0.87	-	0.94(0.60-1.47)	0.78	-	1.03(0.51-2.11)	0.93	-
*CACNG6_BL1_ht2*			0.207	0.328	0.211	0.202	0.142	0.095	0.97(0.67-1.41)	0.88	-	0.93(0.58-1.47)	0.74	-	1.14(0.46-2.80)	0.78	-
*CACNG6_BL1_ht3*			0.153	0.259	0.152	0.154	0.62	0.242	0.94(0.60-1.46)	0.77	-	0.87(0.53-1.43)	0.58	-	1.72(0.42-7.09)	0.45	-
*CACNG6_BL1_ht4*			0.097	0.175	0.083	0.104	0.705	0.472	0.8(0.46-1.38)	0.41	-	0.78(0.43-1.41)	0.41	-	0.77(0.09-6.70)	0.82	-
*CACNG6_BL1_ht5*			0.082	0.151	0.054	0.093	0.565	0.677	0.57(0.29-1.12)	0.1	0.76	0.58(0.29-1.15)	0.12	0.85	.	0.99	-
*CACNG6_BL1_ht6*			0.057	0.107	0.093	0.045	0.3	0.899	2.57(1.39-4.73)	**0.003**	**0.02**	2.81(1.51-5.24)	**0.001**	**0.0087**	.	0.99	-
*CACNG6_BL2_ht1*			0.495	0.5	0.422	0.509	0.447	0.6	0.75(0.55-1.03)	0.07	0.52	0.65(0.40-1.04)	0.07	0.52	0.73(0.42-1.26)	0.26	-
*CACNG6_BL2_ht2*			0.29	0.412	0.338	0.283	0.238	0.538	1.31(0.94-1.83)	0.11	0.77	1.66(1.06-2.60)	**0.03**	0.2	0.93(0.43-2.04)	0.86	-
*CACNG6_BL2_ht3*			0.13	0.226	0.152	0.129	0.785	0.173	1.1(0.69-1.74)	0.68	-	1.07(0.65-1.77)	0.78	-	1.76(0.30-10.15)	0.53	-
*CACNG6_BL2_ht4*			0.068	0.127	0.069	0.063	0.457	0.567	0.93(0.49-1.77)	0.83	-	0.96(0.50-1.84)	0.89	-	.	0.99	-

^a^The frequency of SNP indicates the minor allele frequency.

HWE, Hardy-Weinberg equilibrium; *P^corr^*, corrected *P*-value using multiple testing corrections.

AIA, aspirin-intolerant asthma; ATA, aspirin-tolerant asthma; AA, amino acid; MAF, minor allele frequency; OR, odds ratio; CI, confidence interval

Based on the 8 polymorphisms, haplotypes were calculated using PHASE software resulting in two haplotype blocks (Fig. 1B and 1C). Although a different pattern in Block 1 between AIA and ATA was observed (Fig. 1D), the haplotypes which were composed of five SNPs were used for the association analysis because they were estimated as common haplotypes derived from a large number of subjects in the Korean population. Among the haplotypes, *CACNG6_BL1_ht6*, the only one containing the rare T variant of *rs192808*, also showed significant association with risk of AIA (*P *= 0.003, *P^corr ^*= 0.02 in co-dominant model; *P *= 0.001, *P^corr ^*= 0.0087 in dominant model), showing a similar pattern with the association of *rs192808 *SNP (Table 2). In the case of *CACNG6_BL1_ht6 *haplotype, the frequency of AIA patients was higher at about two-fold than that of ATA controls (0.093 and 0.045, respectively).

Next, we performed regression analysis for the association between decline of FEV_1 _by aspirin provocation and *CACNG6 *polymorphisms. As summarized in Table 3, among all the variants and haplotypes, SNP *rs192808 *(*P *= 0.006 in co-dominant model and *P *= 0.01 in dominant model) and haplotype *CACNG6_BL1_ht6 *(*P *= 0.03 in co-dominant model and *P *= 0.02 in dominant model) showed the most significant signal. In addition, in the second LD block, haplotype *CACNG6_BL2_ht2 *showed an association with the decline of FEV_1 _by aspirin provocation in the dominant model (*P *= 0.03). However, in further regression analysis using only AIA cases, no significant associations were detected (Additional file 1, Table S1), indicating that *rs192808 *and haplotypes of *CACNG6 *could not directly affect the aspirin-induced decline of FEV_1 _in AIA patients.

**Table 3:** Regression analysis between SNPs and haplotypes of *CACNG6 *in all subjects

SNP or Haplotype	Position	C/C	C/R	R/R	*Pa*	*Pb*	*Pc*
*rs251850*	Promoter	397 (9.41 ± 13.26)	180 (8.48 ± 12.24)	15 (13.60 ± 21.54)	0.91	0.57	0.19
*rs4806481*	Intron	153 (10.09 ± 13.84)	308 (9.34 ± 13.47)	129 (8.07 ± 11.90)	0.25	0.38	0.32
*rs158196*	Intron	251 (9.19 ± 13.69)	265 (8.81 ± 12.70)	76 (10.85 ± 13.47)	0.75	0.79	0.29
*rs158199*	Intron	349 (8.92 ± 12.97)	204 (9.63 ± 13.63)	39 (9.97 ± 13.55)	0.75	0.78	0.81
*rs192808*	Intron	529 (8.81 ± 12.99)	60 (12.35 ± 13.94)	3 (21.67 ± 28.36)	**0.006**	**0.01**	0.06
*CACNG6_BL1_ht1*		259 (9.55 ± 13.60)	271 (9.11 ± 12.76)	62 (8.45 ± 13.77)	0.64	0.68	0.73
*CACNG6_BL1_ht2*		382 (9.19 ± 13.48)	178 (9.05 ± 12.56)	32 (10.72 ± 13.99)	0.98	0.82	0.59
*CACNG6_BL1_ht3*		421 (9.34 ± 13.30)	159 (8.41 ± 11.88)	12 (16.39 ± 23.38)	0.92	0.64	0.07
*CACNG6_BL1_ht4*		481 (9.40 ± 13.60)	104 (8.67 ± 11.80)	7 (6.14 ± 2.97)	0.5	0.56	0.57
*CACNG6_BL1_ht5*		493 (9.52 ± 13.23)	96 (7.91 ± 13.32)	3 (4.67 ± 2.31)	0.32	0.33	0.66
*CACNG6_BL1_ht6*		530 (8.83 ± 12.99)	60 (12.90 ± 14.91)	2 (5.50 ± 6.36)	**0.03**	**0.02**	0.69
*rs450227*	Intron	426 (9.18 ± 13.11)	157 (9.38 ± 13.56)	9 (9.33 ± 13.76)	0.89	0.91	0.87
*rs2291068*	Exon	378 (9.17 ± 13.17)	194 (9.40 ± 13.14)	20 (8.72 ± 15.41)	0.69	0.79	0.61
*rs459247*	Exon	150 (11.12 ± 15.52)	290 (8.74 ± 11.28)	152 (8.32 ± 14.07)	0.12	0.06	0.48
*CACNG6_BL2_ht1*		153 (11.09 ± 15.38)	290 (8.70 ± 11.28)	149 (8.37 ± 14.19)	0.12	0.06	0.50
*CACNG6_BL2_ht2*		301 (8.17 ± 12.70)	240 (10.55 ± 13.87)	51 (9.34 ± 12.78)	0.09	**0.03**	0.97
*CACNG6_BL2_ht3*		442 (9.37 ± 13.28)	145 (8.72 ± 12.92)	5 (11.80 ± 18.75)	0.51	0.44	0.75
*CACNG6_BL2_ht4*		515 (9.12 ± 13.08)	76 (9.99 ± 14.29)	1 (8.00)	0.86	0.83	0.78

C/C, common allele/common allele; C/R, common allele/rare allele; R/R, rare allele/rare allele;

*Pa*, *P*-values of co-dominant model; *Pb*, dominant model; *Pc*, recessive model.

## Discussion

Although over-production of pro-inflammatory cysLTs such as LTC4, LTD4 and LTE4 has been considered as a main cause of aspirin hypersensitivity in asthma [25], recent studies have suggested that other genes may be related to AIA [21,26,27]. On the other hand, previous studies have reported that concentration of calcium ion is related with leukotriene secretion [28] and that *CACNG6 *gene is negatively expressed in chronic obstructive pulmonary disease and human airway epithelium following injury [16,17]. Considering these facts, we hypothesized that the modulation of calcium concentration could play an important role in abnormal signaling pathways in the airways and that the *CACNG6 *gene, as a subunit of L-type calcium channels, and its polymorphisms might be associated with risk of AIA.

Although the minor T allele of *rs192808 *and its unique haplotype (*CACNG6_BL1_ht6*) showed significant association signals with decline of FEV_1 _(Table 3), allelic dose effect revealed no consistent results of decline of FEV_1 _between *rs192808 *and *CACNG6_BL1_ht6 *due to the small number of rare alleles (R/R), therefore further replication studies are needed for a correct association. Among polymorphisms in the *CACNG6 *gene, the *rs192808 *polymorphism in intronic region showed a highly significant signal. It has also been found that CACNG6 has three transcripts. Thus, to predict role of *rs192808*, we performed GeneSplicer http://www.cbcb.umd.edu/software/GeneSplicer and EMBL-EBI splice site prediction tool http://www.ebi.ac.uk/asd-srv/wb.cgi?method=2 to know whether *rs192808 *could be located in splicing association site [29]. However, results showed that the *rs192808 *was not located in any splicing-related site such as donor site (5' boundary), acceptor site (3' boundary) and branch point. In addition, the functionality of other SNPs that were in LD with *rs192808 *was analyzed. However, results from the Signal Scan program http://www-bimas.cit.nih.gov/molbio/signal/ revealed that *CACNG6 rs251850 *in promoter region was not a transcriptional element, and three intronic SNPs (*rs4806481*, *rs158196*, *rs158199*) were not predicted as splicing-related sites.

A previous study has reported that some SNPs located deep in intronic region can create new transcription factor binding site [30]. Thus, we further performed TFSEARCH http://www.cbrc.jp/research/db/TFSEARCH.html using bioinformatical software to predict whether *rs192808 *can create transcription binding site. However, results showed that the location of *rs192808 *is not in the transcription factor binding site. Even if these results suggest that *rs192808 *may not affect the function of *CACNG6*, previous studies reported that sometimes allelic variants in intron can lead to the onset of human diseases due to alterations in the expression level of mRNA caused by creating potential aberrant splice sites [31,32]. Moreover, additional transcriptional isoforms of the *CACNG6 *gene have been discovered from the Mammalian Gene Collection (MGC) Program [33] as shown in the Ensembl Genome Browser http://www.ensembl.org/index.html (Additional files 3, Figure S1). Therefore, findings from our study on the significant association between *rs192808 *and AIA needs to be replicated and/or further analyzed in order to understand its detailed function in the mechanism of AIA.

Since there is a possibility that *CACNG6 *SNPs are in LD with functional SNP(s) that is/are located in a different gene in the nearby region, we further analyzed the LD near *CACNG6 *in Asian populations (Japanese and Chinese) from the International HapMap Project. However, the *CACNG6 *gene showed no LD with other nearby genes (Additional files 4, Figure S2). On the other hand, although minor allele frequencies of 8 SNPs validated in Korean asthmatics were similar to those of Asian populations (Chinese and Japanese), significant differences were detected from other populations especially in *rs158196*, *rs1581199*, *rs192808*, *rs450227*, *rs2291068*, and *rs459247 *(Additional files 2, Table S2). When comparing LDs on 8 SNPs of *CACNG6 *among Korean asthmatic patients and other populations (Additional file 5, Figure S3), albeit non-asthmatic Korean population has not determined, it can be assumed that a different LD status of the asthmatics may affect AIA and/or asthma-related phenotypes at least in a Korean population. Moreover, although replications and functional studies are needed, *rs192808 *and *CACNG6_BL1_ht6 *could be genetic markers of AIA susceptibility.

## Conclusions

Although replication for the associations is required in an independent study cohort to validate our findings, this study showed that the genetic polymorphisms of *CACNG6 *might be associated with risk of AIA at least in a Korean population, providing a new link between voltage-dependent calcium channel and aspirin hypersensitivity in asthmatics. Further functional and replication studies elsewhere are needed to identify the roles of the polymorphisms of the gene.

## List of Abbreviations

AIA: aspirin-intolerant asthma; ATA: aspirin-tolerant asthma; CACNG6: calcium channel: voltage-dependent: gamma subunit 6; SNP: single nucleotide polymorphism; MAF: minor allele frequency; LD: linkage disequilibrium; OR: odds ratio; CI: confidence interval

## Competing interests

The authors declare that they have no competing interests.

## Authors' contributions

JSL developed tables/figures, and drafted the manuscript. JK, JSB and CFP helped to interpret the data and to draft the manuscript. SU, JP, AJ, MK, ISC and CP recruited subjects. TJP and JYK, participated in preparation and quality control of samples, and data collection. BP and HSC performed the statistical analysis. CP and HDS coordinated all of this study and helped to draft the manuscript. All authors read and approved the final manuscript.

## Pre-publication history

The pre-publication history for this paper can be accessed here:

http://www.biomedcentral.com/1471-2350/11/138/prepub

## Supplementary Material

Additional file 1 **Regression analysis between SNPs and haplotypes of *CACNG6 *in patients with AIA only**. Results of regression analysis in AIA patients only Click here for file

Additional file 2 **Allele information in other population for *CACNG6***. Allele frequencies of *CACNG6 *polymorphisms among Korean and other populations Click here for file

Additional file 3 **Three isoforms of *CACNG6***. Three different types of the *CACNG6 *gene transcripts are found from the Ensembl Genome Browser http://www.ensembl.org/index.html. Click here for file

Additional file 4 **LD plot nearby *CACNG6***. LD blocks nearby CACNG6 based on Asian populations (Japanese and Chinese) show no LD between *CACNG6 *and near genes. (A) LD blocks of near genes and *CACNG6*. Data for LD map is obtained from the International HapMap project. (B) LD block of the *CACNG6 *gene. Click here for file

Additional file 5 LDs of *CACNG6 *polymorphisms among populations. Comparison of LDs of *CACNG6 *between Korean asthmatics and other populations. (A) Korean asthmatics. (B) Korean AIA. (C) Korean ATA. (D) Caucasian. (E) Asians including Japanese and Chinese. (F) Africans Click here for file
