# Supplementary material for: Association of CACNG6 polymorphisms with aspirin-intolerance asthmatics in a Korean population
Source: BMC Med Genet. 2010 Sep 23;11:138. doi: 10.1186/1471-2350-11-138 (PMC2954844; doi:10.1186/1471-2350-11-138)
Supplement: Additional file 1 — Regression analysis between SNPs and haplotypes of CACNG6 in patients with AIA only. Results of regression analysis in AIA patients only [file 1471-2350-11-138-S1.DOC]

**Supplementary Table 1: Regression analysis between SNPs and haplotypes of *CACNG6*** in patients with AIA only

| SNP or Haplotype | Position | C/C | C/R | R/R | *Pa* | *Pb* | *Pc* |
| --- | --- | --- | --- | --- | --- | --- | --- |
| *rs251850 T>C* | Promoter | 68(33.56±13.22) | 28(32.32±11.25) | 3(46.33±31.56) | 0.55 | 0.96 | 0.09 |
| *rs4806481 C>T* | Intron1 | 28(33.56±14.82) | 52(34.53±12.52) | 19(31.09±14.05) | 0.56 | 0.93 | 0.35 |
| *rs158196 A>G* | Intron1 | 42(34.27±14.40) | 43(32.50±13.37) | 14(34.94±10.90) | 0.89 | 0.67 | 0.74 |
| *rs158199 G>A* | Intron2 | 56(33.44±13.42) | 35(34.15±14.41) | 8(32.26±9.62) | 0.97 | 0.90 | 0.76 |
| *rs192808 C>T* | Intron3 | 80(34.18±13.56) | 18(29.87±11.93) | 1(54.00) | 0.72 | 0.44 | 0.11 |
| *CACNG6_BL1_ht1* |  | 44(34.27±14.19) | 44(33.23±12.39) | 11(32.34±15.32) | 0.59 | 0.59 | 0.77 |
| *CACNG6_BL1_ht2* |  | 64(34.26±13.83) | 28(32.33±13.44) | 7(32.59±10.35) | 0.55 | 0.51 | 0.82 |
| *CACNG6_BL1_ht3* |  | 73(33.41±13.13) | 23(32.52±11.15) | 3(46.33±31.56) | 0.44 | 0.81 | 0.09 |
| *CACNG6_BL1_ht4* |  | 84(33.81±13.64) | 15(32.36±12.45) | . | 0.66 | 0.66 | . |
| *CACNG6_BL1_ht5* |  | 88(32.87±13.24) | 11(39.35±14.06) | . | 0.14 | 0.14 | . |
| *CACNG6_BL1_ht6* |  | 81(34.01±13.56) | 18(31.70±12.98) | . | 0.59 | 0.59 | . |
| *rs450227 C>T* | Intron3 | 68(34.05±13.79) | 30(32.19±12.74) | 1(45.00) | 0.76 | 0.63 | 0.45 |
| *rs2291068 T>C* | Exon4 | 60(34.05±14.53) | 34(33.22±11.73) | 5(30.68±12.18) | 0.61 | 0.74 | 0.54 |
| *rs459247 G>A* | Exon4 | 34(36.19±12.35) | 45(29.65±11.10) | 20(38.04±17.58) | 0.93 | 0.16 | 0.11 |
| *CACNG6_BL2_ht1* |  | 35(35.61±12.64) | 44(29.96±11.03) | 20(38.04±17.58) | 0.92 | 0.27 | 0.11 |
| *CACNG6_BL2_ht2* |  | 41(33.84±14.37) | 49(33.50±13.11) | 9(33.00±11.92) | 0.93 | 0.95 | 0.93 |
| *CACNG6_BL2_ht3* |  | 71(34.57±13.78) | 27(30.59±12.28) | 1(45.00) | 0.37 | 0.26 | 0.45 |
| *CACNG6_BL2_ht4* |  | 85(33.19±14.03) | 14(36.03±8.80) | . | 0.44 | 0.44 | . |

C/C, common allele/common allele; C/R, common allele/rare allele; R/R, rare allele/rare allele.

*Pa*, *P*-values of co-dominant model; *Pb*, dominant model; *Pc*, recessive model.
