# Supplementary material for: Association of CACNG6 polymorphisms with aspirin-intolerance asthmatics in a Korean population
Source: BMC Med Genet. 2010 Sep 23;11:138. doi: 10.1186/1471-2350-11-138 (PMC2954844; doi:10.1186/1471-2350-11-138)
Supplement: Additional file 2 — Allele information in other population for CACNG6. Allele frequencies of CACNG6 polymorphisms among Korean and other populations [file 1471-2350-11-138-S2.DOC]

***Supplementary Table 2: Allele information in other population for*** CACNG6

| rs # | Allele | Ref. Allele |  | Minor allele frequency | | | | |
| --- | --- | --- | --- | --- | --- | --- | --- | --- |
|  |  |  |  | KOR | CEU | CHB | JPT | YRI |
| *rs251850* | C/T | T |  | 0.18 | 0.26 | 0.2 | 0.15 | 0.17 |
| *rs4806481* | C/T | C |  | 0.48 | 0.37 | 0.47 | 0.49 | NA |
| *rs158196* | A/G | A |  | 0.35 | 0.16 | 0.28 | 0.44 | 0.44 |
| *rs158199* | A/G | G |  | 0.24 | 0.12 | 0.19 | 0.25 | 0.34 |
| *rs192808* | A/G | A |  | 0.06 | 0.26 | 0.07 | 0.04 | 0.2 |
| *rs450227* | A/G | G |  | 0.15 | 0.02 | 0.11 | 0.19 | NA |
| *rs2291068* | A/G | A |  | 0.20 | 0.02 | 0.09 | 0.23 | NA |
| *rs459247* | C/T | C |  | 0.50 | 0.37 | 0.45 | 0.44 | 0.14 |

KOR, Korean asthmatics; CEU, Caucasian; CHE, Chinese; JPT, Japanese; YRI, African. NA, not available.

This table established using international HapMap project data (http://hapmap.ncbi.nlm.nih.gov).
