# Supplementary figures and images for: Association of CACNG6 polymorphisms with aspirin-intolerance asthmatics in a Korean population
Source: BMC Med Genet. 2010 Sep 23;11:138. doi: 10.1186/1471-2350-11-138 (PMC2954844; doi:10.1186/1471-2350-11-138)

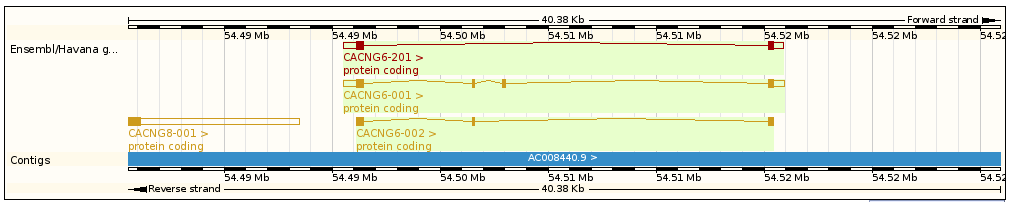

Supplement: Additional file 3 — Three isoforms of CACNG6. Three different types of the CACNG6 gene transcripts are found from the Ensembl Genome Browser http://www.ensembl.org/index.html. [file 1471-2350-11-138-S3.TIFF]

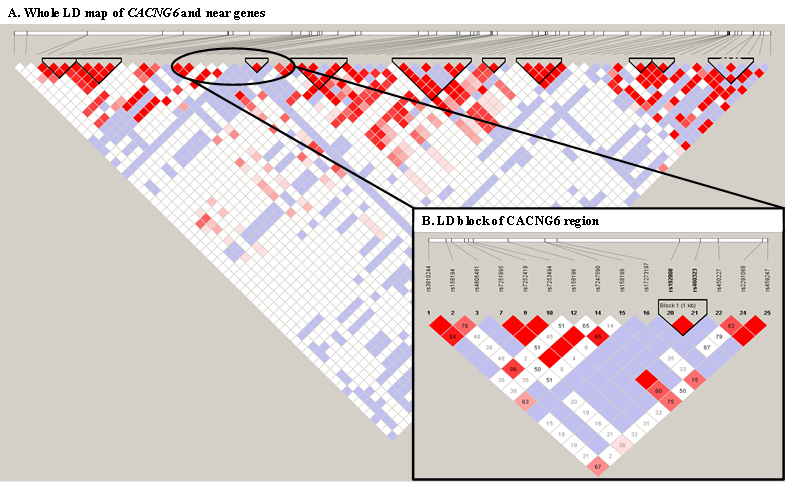

Supplement: Additional file 4 — LD plot nearby CACNG6. LD blocks nearby CACNG6 based on Asian populations (Japanese and Chinese) show no LD between CACNG6 and near genes. (A) LD blocks of near genes and CACNG6. Data for LD map is obtained from the International HapMap project. (B) LD block of the CACNG6 gene. [file 1471-2350-11-138-S4.TIFF]

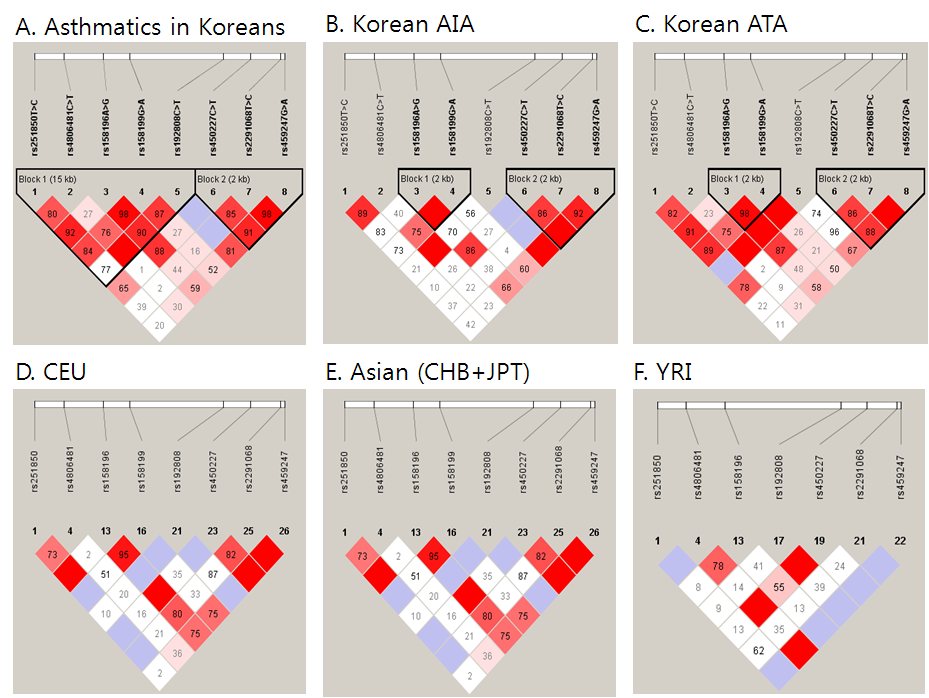

Supplement: Additional file 5 — LDs of CACNG6 polymorphisms among populations. Comparison of LDs of CACNG6 between Korean asthmatics and other populations. (A) Korean asthmatics. (B) Korean AIA. (C) Korean ATA. (D) Caucasian. (E) Asians including Japanese and Chinese. (F) Africans [file 1471-2350-11-138-S5.TIFF]
